# Spinal Central Effects of Peripherally Applied Botulinum Neurotoxin A in Comparison between Its Subtypes A1 and A2

**DOI:** 10.3389/fneur.2014.00098

**Published:** 2014-06-23

**Authors:** Hidetaka Koizumi, Satoshi Goto, Shinya Okita, Ryoma Morigaki, Norio Akaike, Yasushi Torii, Tetsuhiro Harakawa, Akihiro Ginnaga, Ryuji Kaji

**Affiliations:** ^1^Department of Clinical Neuroscience, Institute of Health Biosciences, Graduate School of Medical Sciences, University of Tokushima, Tokushima, Japan; ^2^Department of Motor Neuroscience and Neurotherapeutics, Institute of Health Biosciences, Graduate School of Medical Sciences, University of Tokushima, Tokushima, Japan; ^3^Research Division for Life Science, Kumamoto Health Science University, Kumamoto, Japan; ^4^The Chemo-Sero-Therapeutic Research Institute (KAKETSUKEN), Kumamoto, Japan; ^5^Graduate School of Medicine, Osaka University, Osaka, Japan

**Keywords:** botulinum neurotoxin, spinal cord, central effects, SNAP-25, axonal transport

## Abstract

Because of its unique ability to exert long-lasting synaptic transmission blockade, botulinum neurotoxin A (BoNT/A) is used to treat a wide variety of disorders involving peripheral nerve terminal hyperexcitability. However, it has been a matter of debate whether this toxin has central or peripheral sites of action. We employed a rat model in which BoNT/A1 or BoNT/A2 was unilaterally injected into the gastrocnemius muscle. On time-course measurements of compound muscle action potential (CMAP) amplitudes after injection of BoNT/A1 or BoNT/A2 at doses ranging from 1.7 to 13.6 U, CMAP amplitude for the ipsilateral hind leg was markedly decreased on the first day, and this muscle flaccidity persisted up to the 14th day. Of note, both BoNT/A1 and BoNT/A2 administrations also resulted in decreased CMAP amplitudes for the contralateral leg in a dose-dependent manner ranging from 1.7 to 13.6 U, and this muscle flaccidity increased until the fourth day and then slowly recovered. Immunohistochemical results revealed that BoNT/A-cleaved synaptosomal-associated protein of 25 kDa (SNAP-25) appeared in the bilateral ventral and dorsal horns 4 days after injection of BoNT/A1 (10 U) or BoNT/A2 (10 U), although there seemed to be a wider spread of BoNT/A-cleaved SNAP-25 associated with BoNT/A1 than BoNT/A2 in the contralateral spinal cord. This suggests that the catalytically active BoNT/A1 and BoNT/A2 were axonally transported via peripheral motor and sensory nerves to the spinal cord, where they spread through a transcytosis (cell-to-cell trafficking) mechanism. Our results provide evidence for the central effects of intramuscularly administered BoNT/A1 and BoNT/A2 in the spinal cord, and a new insight into the clinical effects of peripheral BoNT/A applications.

## Introduction

Botulinum neurotoxins (BoNTs) have traditionally been subdivided into eight distinguishable serotypes (types A through H) (1, 2). They are potent poisons that disrupt neurotransmission by their proteolytic activity directed specifically at SNARE (soluble *N*-ethylmaleimide-sensitive fusion protein attachment receptor) proteins, which are essential for synaptic vesicle fusion and transmitter release (1). Because of their long-lasting effects, botulinum neurotoxin type A (BoNT/A) and BoNT/B are currently used in a broad variety of therapeutic interventions (3, 4), such as for spasticity (5), movement disorders (6), and pathological pain conditions (7). BoNT/A is a metalloprotease that targets and cleaves synaptosomal-associated protein of 25 kDa (SNAP-25), a member of the SNARE family, thereby blocking the release of neurotransmitters (e.g., acetylcholine) from peripheral nerve terminals (8–10). BoNT/A has been serologically classified into seven subtypes (A1–A7), in which neurotoxin components vary in their amino acid sequences (11–13), and its A1 subtype (BoNT/A1) is currently used in clinics.

There is a traditional view that BoNT/A effects remain localized to peripheral neuromuscular junctions near the toxin injection site. However, it is becoming clear that some of BoNT/A1’s clinical effects cannot be explained without assuming direct central effects (4, 14, 15), and a potential central site of action has been a matter of debate. In addition, the biological effects of the BoNT/A subtypes other than BoNT/A1 are poorly understood. To address these issues experimentally, we employed a rat model in which either BoNT/A1 or BoNT/A2 was injected into the gastrocnemius muscle of a hind leg. Our results provide evidence for the central actions of catalytically active BoNT/A1 and BoNT/A2 via axonal and transsynaptic transport from the periphery into the spinal cord, where they spread via a transcytosis (cell-to-cell trafficking) mechanism.

## Materials and Methods

### Animals and ethics statement

Sprague Dawley rats aged 8 weeks (180–220 g; Charles River Laboratories Japan, Yokohama, Japan) were used. The rats were housed in a controlled environment (25 ± 1°C, 50 ± 10% humidity, and 12-h light/dark cycle) with access to food and tap water *ad libitum*. All procedures involving experimental animals were approved by the Ethical Review Committee of the University of Tokushima and the Chemo-Sero-Therapeutic Research Institute (KAKETSUKEN).

### Purification of toxins

BoNT/As were prepared employing a previously reported method (16) with minor modifications (17). Briefly, *Clostridium botulinum* type A strains 62A and Chiba-H, which belong to subtypes A1 and A2, respectively, were cultured in a PYG medium, containing 2% peptone, 0.5% yeast extract, 0.5% glucose, and 0.025% sodium thioglycolate, at 30°C for 3 days. M toxin was purified from the culture fluid by acid precipitation, protamine treatment, ion-exchange chromatography, and gel filtration. Each subtype of M toxin was adsorbed onto a DEAE Sepharose column equilibrated with 10 mM phosphate buffer, and eluted with a 0–0.3 M NaCl gradient buffer to separate BoNT/A and non-toxic components. The different types of BoNT/A were stored at −70°C until use.

### Toxic activity measurements

The toxic activities of BoNT/A1 and BoNT/A2 were determined by employing the mouse intraperitoneal (i.p.) LD_50_ method, as described previously (17). One mouse i.p. LD_50_ was defined as 1 unit (U).

### Measurements of compound muscle action potentials

Compound muscle action potential (CMAP) measurements were performed according to the method that we previously reported (18). Rats were anesthetized with pentobarbital (Kyoritsu Seiyaku, Tokyo, Japan) and fixated in the prone position. The electrode used was an alligator clip lead wire (Viasys Healthcare, Tokyo, Japan) attached to the skin. The stimulating electrodes (cathodes) were placed on the skin over the fourth lumbar vertebra, and the stimulating electrodes (anodes) were placed at 2 cm from the cathode on the spinal column. The recording electrodes were placed on the belly muscles of the left and right gastrocnemius muscles, the reference recording electrodes on the left gastrocnemius tendons, and the earth electrodes on the tail roots. Electric stimulation was loaded at 25 mA for 0.2 ms, and the CMAP was measured using a Nicolet Viking Quest EMG system (Viasys Healthcare, Tokyo, Japan).

### Western blots

Rats were sacrificed 4 days after stereotactic injection of BoNT/A1 (20 U) (*n* = 3) or saline (*n* = 3) into the unilateral striatum. The striatal lysates were prepared and subjected to western blot analysis, as previously reported by Yamamura et al. (19). Briefly, striatal tissue samples were homogenized in ice-cold lysis buffer containing 50 mM Tris–HCl buffer, pH 7.5, with 0.5 M NaCl, 0.5% Triton X-100, 10 mM EDTA, 4 mM EGTA, 1 mM Na_3_VO_4_, 30 mM Na_2_P_2_O_7_, 50 mM NaF, 0.1 mM leupeptin, 75 μM pepstatin A, 50 μg/ml trypsin inhibitor, 1 mM phenylmethanesulfonyl fluoride, 100 nM calyculin A, and 1 mM dithiothreitol. After centrifugation at 15,000 rpm for 10 min at 4°C, the protein lysates were mixed with Laemmli buffer containing 63 mM Tris–HCl (pH 6.8), 2% sodium dodecylsulfate (SDS), 5% 2-mercaptoethanol, 2.5% glycerol, and 0.01% bromophenol blue, and were then heated at 100°C for 5 min. Each sample, containing the same amount of proteins (20 μg/lane), was applied to a 12% SDS–polyacrylamide gel electrophoresis (PAGE) followed by blotting onto a PVDF membrane. The PVDF membranes were then incubated with the desired primary antibodies. Monoclonal antibody (mAb) against BoNT/A-cleaved SNAP-25 (1:1,000; GENTAUR Molecular, Santa Clara, CA, USA) and polyclonal antibody against SNAP-25 protein (1:10,000; GeneTex, Irvine, CA, USA) were used.

### Immunohistochemical staining and digital imaging

Rats were sacrificed 4 days after unilateral injection of BoNT/A1 (10 U; *n* = 10), BoNT/A2 (10 U; *n* = 10), or saline (*n* = 3) into the left gastrocnemius muscles. Deeply anesthetized rats were transcardially perfused with 0.01 M phosphate-buffered saline (pH 7.4) (PBS), followed by 4% paraformaldehyde in 0.1 M phosphate buffer (pH 7.4). After laminectomy, the spinal cords at the lumbosacral region were removed. They were post-fixed for 12 h in the same fixative, and stored in a 10–30% sucrose gradient in 0.1 M phosphate buffer at 4°C. Frozen sections with 25 μm-thickness were prepared on a cryostat, and then stored in PBS containing 0.05% NaN_3_ until use. For single antigen detection, free-floating sections were pretreated with 1.0% hydrogen peroxide for 15 min. As a blocking step, sections were then incubated in PBS containing 3% bovine serum albumin (BSA) and 50% normal goat serum (NGS) for 3 h at room temperature. This was followed by incubation in primary antibody diluted in PBS containing 3% BSA and 50% NGS overnight at room temperature. The primary antibodies used were mouse mAb against BoNT/A-cleaved SNAP-25 (1:5,000; GENTAUR Molecular) or rabbit polyclonal antibody against SNAP-25 (1:20,000; GeneTex). Bound antibodies were visualized with the Histofine Simple Stain Kit (Nichirei, Tokyo, Japan) and the tyramide signal amplification (TSA) system with Cyanine 3 or Fluorescein (Perkin Elmer LAS), according to the methods reported previously (20, 21). For dual antigen detection, the sections stained for BoNT/A-cleaved-SNAP-25 were incubated in 0.1 M glycine–HCl (pH 2.2) at room temperature for 30 min. After rinsing in PBS for 1 h, they were then incubated overnight at room temperature in PBS containing 3% BSA and polyclonal antibody against choline acetyltransferase (ChAT) (1:20,000; Millipore). The bound antibodies were detected by the Histofine Simple Stain Kit (Nichirei) and the TSA system with Fluorescein (Perkin Elmer). Digital microscopy images were captured using an Olympus BX51 microscope (Olympus, Tokyo, Japan), imported into Adobe Photoshop CS4, and processed digitally for adjustments of contrast, brightness, and color balance.

### Densitometric analysis

To estimate the density of BoNT/A-cleaved SNAP-25 labeling, the immunostaining of the L5 spinal sections from rats that received saline, BoNT/A1 or BoNT/A2 was simultaneously carried out in parallel using the same protocols. By means of Meta Morph (Meta Imaging Series 7.0; Molecular Devices, Tokyo, Japan), the optical densities of immunoreactive products were measured on the raw digital images of the ventral horns of the spinal cord. For each animal that received saline (*n* = 3), BoNT/A1 (10 U; *n* = 6), or BoNT/A2 (10 U; *n* = 6), measurements were made in the ventral horns of three spinal sections.

### Statistical analysis

All experimental values were expressed as means ± SD. Statistical significance was evaluated by one-way ANOVA followed by the Games–Howell *post hoc* test for pairwise comparisons, or by the Mann–Whitney *U*-test. The significance level was set at *P* < 0.05.

## Results

### Biological effects of intramuscularly injected BoNT/A1 and BoNT/A2

**Figure 1:**
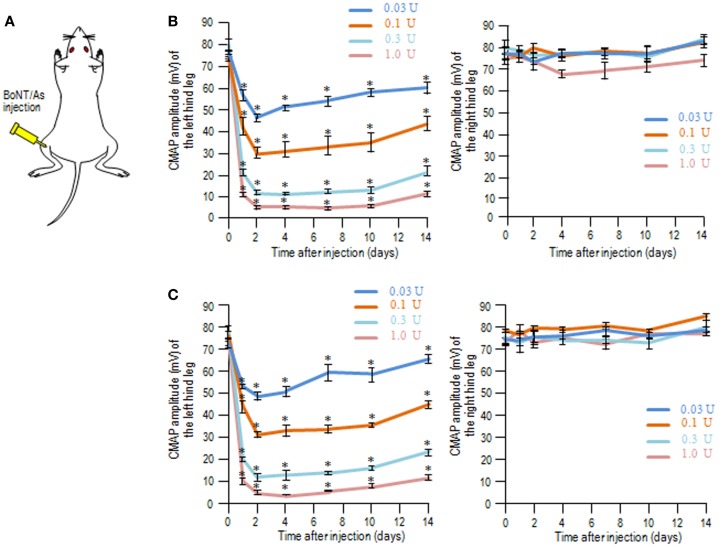
**Compound muscle action potential measurements following unilateral intramuscular injection of BoNT/A1 or BoNT/A2 at lower doses**. **(A)** Experimental protocol. BoNT/A1 or BoNT/A2 was unilaterally injected into the left gastrocnemius muscle, and the CMAP amplitudes of both the hind legs were measured over time. **(B,C)** Time-sequential changes in the CMAP amplitudes of left and right hind legs after peripheral BoNT/A1 **(B)** or BoNT/A2 **(C)** injections at lower doses of 0.03, 0.1, 0.3, or 1.0 U. Each point is the mean ± SD (*n* = 5). *P < 0.05 versus Day 0 in each dose of toxin; one-way ANOVA followed by Games–Howell *post hoc* test.

We used the CMAP measurements to determine the muscle flaccidity obtained with BoNT/As in rats. BoNT/A1 or BoNT/A2 toxin solution was injected into the left gastrocnemius muscle (Figure 1A), and then the CMAP amplitudes of the left (ipsilateral) and right (contralateral) hind legs were measured over time for 14 days. On time-course measurement of CMAP amplitudes after injection of BoNT/A1 (Figure 1B) or BoNT/A2 (Figure 1C) at lower doses ranging from 0.03 to 1.0 U, we found that CMAP amplitude for the ipsilateral, but not contralateral, hind leg decreased in a dose-dependent manner, and this muscle flaccidity increased until the second day, after which it gradually recovered.

**Figure 2:**
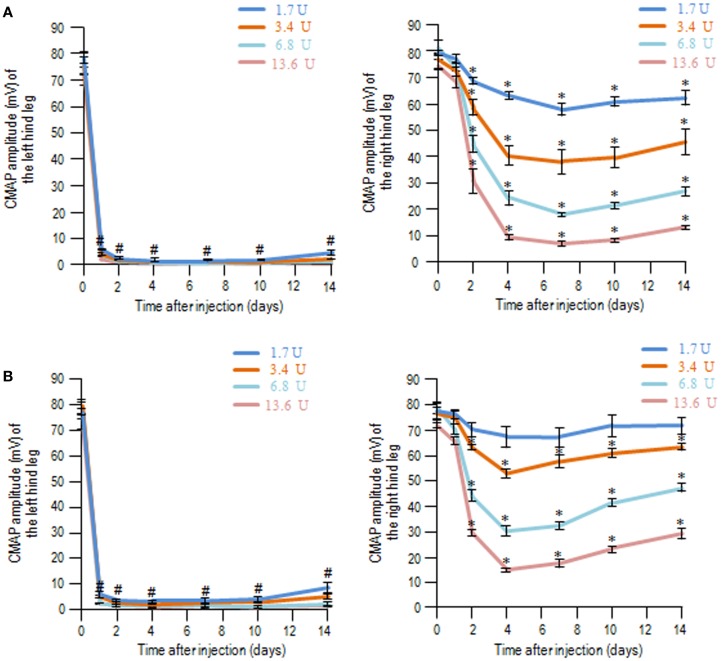
**Compound muscle action potential measurements following unilateral intramuscular injection of BoNT/A1 or BoNT/A2 at higher doses**. Time-sequential changes in the CMAP amplitudes of left and right hind legs after peripheral BoNT/A1 **(A)** or BoNT/A2 **(B)** injections at higher doses of 1.7, 3.4, 6.8, or 13.6 U. Each point is the mean ± SD (*n* = 5). ^#^*P* < 0.001, *P < 0.05 versus Day 0 in each dose of toxin; one-way ANOVA followed by Games–Howell *post hoc* test.

With the same experimental protocols, time-course measurements of CMAP amplitudes after injection of BoNT/A1 (Figure 2A) or BoNT/A2 (Figure 2B) at higher doses ranging from 1.7 to 13.6 U showed that CMAP amplitude for the ipsilateral hind leg markedly decreased on the first day, and this muscle flaccidity persisted up to the 14th day. Interestingly, either BoNT/A1 (Figure 2A) or BoNT/A2 (Figure 2B) administration also caused decreased CMAP amplitudes for the contralateral legs in a dose-dependent manner ranging from 1.7 to 13.6 U, and this muscle flaccidity increased until the fourth day and then slowly recovered. When equivalent doses were assessed, the degree of contralateral muscle flaccidity obtained with BoNT/A2 seemed to be slightly lower than that with BoNT/A1 at each time period. Thus, the biological effects of BoNT/A1 and BoNT/A2 were found in both hind legs only when higher doses of the toxins were used, suggesting that their effects could extend beyond the periphery to affect the contralateral leg.

### Appearance of BoNT/A-cleaved SNAP-25 in the spinal cord after intramuscular injection of BoNT/A1 or BoNT/A2

**Figure 3:**
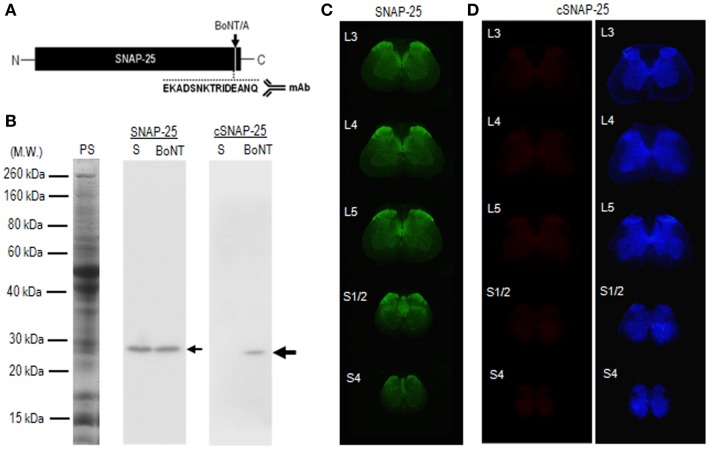
**Characterization of mAb against BoNT/A-cleaved SNAP-25 (cSNAP-25)**. **(A)** Schematic representation of BoNT/A cleavage site (arrow) in the SNAP-25 protein. Specific mAb was raised against the synthetic peptide (EKADSNKTRIDEANQ), which corresponds to the COOH-terminus of the cSNAP-25 protein. **(B)** Specificity of mAb against cSNAP-25 as determined by western blot. Striatal lysates were prepared from rats that received stereotactic injection of BoNT/A1 (20 U; BoNT) or saline (S) into the striatum 4 days before sacrifice. They were then subjected to 12% SDS-PAGE followed by western blot analysis with anti-SNAP-25 or anti-cSNAP-25 antibody (see Materials and Methods). Twenty micrograms of protein were loaded on each lane. Protein staining (PS) and immunostaining for SNAP-25 and cSNAP-25 are shown. Small arrow indicates SNAP-25 protein with an apparent molecular weight of 25 kDa. Large arrow indicates cSNAP-25 protein with an apparent molecular weight of 24 kDa. Note that mAb against cSNAP-25 reacts with the cleaved, but not non-cleaved, form of SNAP-25 protein. **(C)** Multiple transverse spinal sections stained for SNAP-25 from saline-treated rats. **(D)** Multiple transverse spinal cord sections stained for cSNAP-25 from saline-treated rats (left panel), and their graded color-converted images (right panel).

To test whether catalytically active BoNT/A1 and BoNT/A2 actually reach the spinal cord after peripheral intramuscular injection of the toxins, we performed immunohistochemical examinations of spinal cord tissues 4 days after injection of BoNT/A1 (10 U) or BoNT/A2 (10 U) toxin solution into the left gastrocnemius muscles. For this purpose, we used mAb against the synthetic peptide (EKADSNKTRIDEANQ) (Figure 3A), which corresponds to the COOH-terminus of BoNT/A-truncated SNAP-25 protein (22). Western blot analysis (Figure 3B) revealed that the mAb used here reacted with the cleaved form of SNAP-25 but not intact SNAP-25 protein. Immunohistochemical results with the TSA techniques showed significant immunoreactivity for SNAP-25 (Figure 3C), but not cSNAP-25 (Figure 3D), in the spinal cord in the saline-treated control rats. By contrast, we successfully detected cSNAP-25 immunoreactivity in the spinal cord in the toxin-treated rats, as follows.

**Figure 4:**
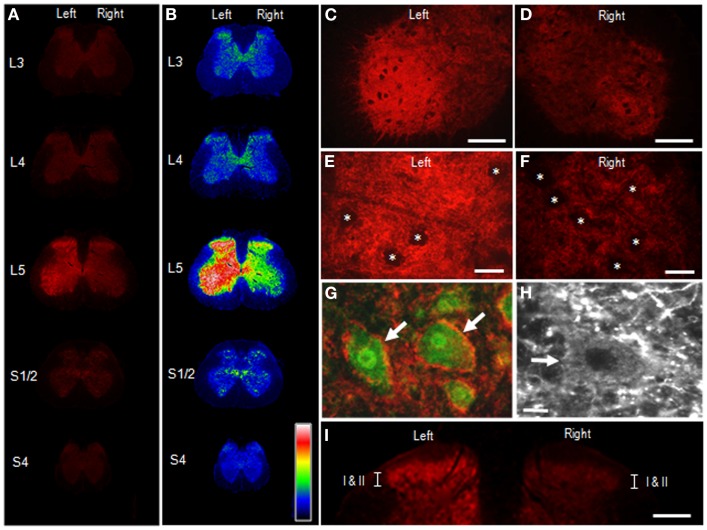
**Appearance of cSNAP-25 in the spinal cord after intramuscular injection of BoNT/A1**. Immunohistochemical detection of cSNAP-25 was carried out in the spinal cord 4 days after unilateral injection of BoNT/A1 (10 U) into the left gastrocnemius muscle. **(A,B)** Displayed are multiple transverse spinal cord sections stained for cSNAP-25 in rats that received BoNT/A1 **(A)**, and their graded color-converted images **(B)**, in which labeling intensity is indicated in a standard pseudocolor scale from blue (lowest level) through green, yellow, red, and white (highest level). **(C,D)** Photomicrographs of the ventral horns stained for cSNAP-25 ipsilateral **(C)** and contralateral **(D)** to peripheral toxin injection. Scale bars = 200 μm. **(E,F)** Photomicrographs of the ventral horns (lamina IX) stained for ipsilateral **(E)** and contralateral **(F)** to peripheral toxin injection. Asterisks indicate spinal motoneurons. Scale bars = 50 μm. **(G)** Photomicrograph of spinal motoneurons (arrows) doubly stained for ChAT (green) and cSNAP-25 (red) ipsilateral to peripheral toxin injection. Scale bar = 25 μm. **(H)** High power-magnified photomicrograph with a longer exposure time showing a motoneuron (arrow) that exhibits cSNAP-25-immunoreactive puncta in its soma. Scale bar = 10 μm. **(I)** Photomicrograph of the dorsal horns labeled for cSNAP-25, which contain lamina I and II (I/II). Scale bar = 400 μm.

Figure 4 illustrates the distributional profiles of cSNAP-25 immunoreactivity in the spinal cord of rats that received BoNT/A1. Macroscopic images of multiple segmental levels of the spinal cord stained for cSNAP-25 are shown in Figures 4A,B. In accordance with the fact that the L5 nerve dominantly innervates the gastrocnemius muscle (23), strong immunoreactivity for cSNAP-25 was observed in the ventral and dorsal horns of the spinal cord at the segmental level of L5 ipsilateral to the peripheral toxin injection site, but also to a lesser extent on the contralateral side. Microscopic observations at higher magnification showed the characteristic localization patterns of cSNAP-25 immunolabeling in the anterior horn (lamina IX) of the spinal cord at L5, where cSNAP-25-immunoreactive products appeared as tiny dots that formed fibrous configurations and were numerously found on the ipsilateral side (Figures 4C,E), but less numerously on the contralateral side (Figures 4D,F). They appeared to delineate the cell bodies of motoneurons labeled for ChAT, an enzyme that catalyzes acetylcholine synthesis (Figures 4G,H). Additionally, cSNAP-25-immunoreactive puncta were also found within the soma of some motoneurons (Figure 4H). In the dorsal horns (Figure 4I), strong cSNAP-25 immunolabeling was seen in the ipsilateral superficial layers (lamina I–II), but also to a lesser extent on the contralateral side.

**Figure 5:**
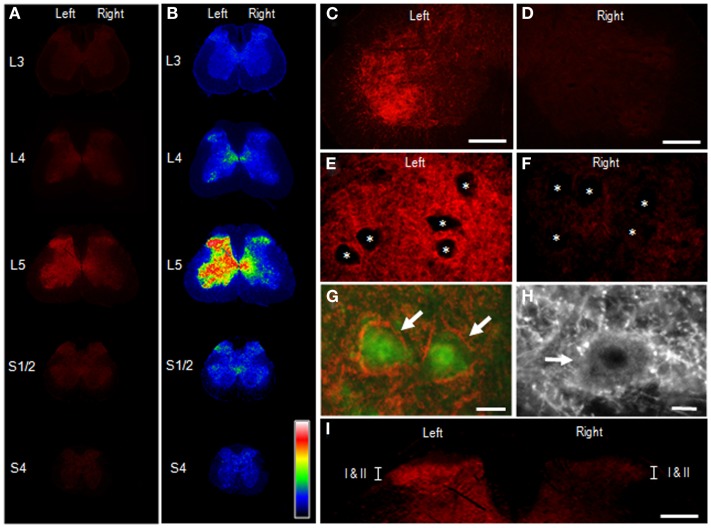
**Appearance of cSNAP-25 in the spinal cord after intramuscular injection of BoNT/A2**. Immunohistochemical detection of cSNAP-25 was carried out in the spinal cord 4 days after unilateral injection of BoNT/A2 (10 U) into the left gastrocnemius muscle. **(A,B)** Displayed are multiple transverse spinal cord sections stained for cSNAP-25 in rats that received BoNT/A2 **(A)**, and their graded color-converted images **(B)**, in which labeling intensity is indicated in a standard pseudocolor scale from blue (lowest level) through green, yellow, red, and white (highest level). **(C,D)** Photomicrographs of the ventral horns stained for cSNAP-25 ipsilateral **(C)** and contralateral **(D)** to peripheral toxin injection. Scale bars = 200 μm. **(E,F)** Photomicrographs of the ventral horns (lamina IX) stained for ipsilateral **(E)** and contralateral **(F)** to peripheral toxin injection. Asterisks indicate spinal motoneurons. Scale bars = 50 μm. **(G)** Photomicrograph of spinal motoneurons (arrows) doubly stained for ChAT (green) and cSNAP-25 (red) ipsilateral to peripheral toxin injection. Scale bar = 25 μm. **(H)** High power-magnified photomicrograph with a longer exposure time showing a motoneuron (arrow) that exhibits cSNAP-25-immunoreactive puncta in its soma. Scale bar = 10 μm. **(I)** Photomicrograph of the dorsal horns labeled for cSNAP-25, which contain lamina I and II (I/II). Scale bar = 400 μm.

Figure 5 illustrates the distributional profiles of cSNAP-25 immunolabeling in the spinal cord of rats that received BoNT/A2. Macroscopic images of multiple segmental levels of the spinal cord stained for cSNAP-25 are shown in Figures 5A,B. As in BoNT/A1-treated rats, strong immunoreactivity for cSNAP-25 was observed in the ventral and dorsal horns at the L5 spinal segment ipsilateral to the peripheral toxin injection site, but also to a lesser extent on the contralateral side. However, there seemed to be a narrower spread of cSNAP-25 associated with BoNT/A2 than BoNT/A1 in the contralateral spinal cord, particularly in the ventral horn. Microscopic observations on the ventral horn (lamina IX) of the spinal cord at L5 showed that cSNAP-25-immunoreactive products appeared as tiny dots that formed fibrous configurations and that they were numerously found on the ipsilateral side (Figures 5C,E), but only sparsely on the contralateral side (Figures 5D,F). They often delineated the cell bodies of motoneurons labeled for ChAT (Figures 5G,H), and cSNAP-25-immunoreactive puncta were also found within the soma of some motoneurons (Figure 5H). In the dorsal horns (Figure 5I), strong cSNAP-25 immunolabeling was seen in the ipsilateral superficial layers (lamina I/II), but also to a lesser extent on the contralateral side.

**Figure 6:**
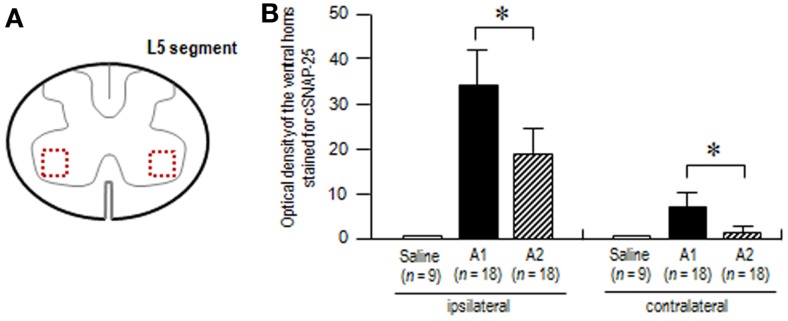
**Densitometric analysis on the spinal ventral horns stained for cSNAP-25**. The optical densities of cSNAP-25-immunoreactive products were measured in the spinal cord at the L5 segmental level 4 days after unilateral injection of saline, BoNT/A1 (10 U) or BoNT/A2 (10 U) into the left gastrocnemius muscle. **(A)** The scheme shows the transverse spinal cord section at the L5 segment, in which measured areas in the bilateral ventral horn are indicated by dashed open boxes colored in red. **(B)** Optical densities of the ventral horns stained for cSNAP-25 in rats treated with saline (*n* = 3), BoNT/A1 (A1) (*n* = 6), or BoNT/A2 (A2) (*n* = 6). For each animal, measurements were made in the ventral horns of three spinal cord sections ipsilateral and contralateral to the toxin-injected sites. Values are means ± SD *P < 0.05, A1 versus A2; Mann–Whitney*U*-test.

To test our assumption that axonal and transsynaptic transport of BoNT/A1 might be greater than that of BoNT/A2 (24), we also carried out the densitometric analysis on the ventral horns stained for cSNAP-25 at the L5 spinal segment in rats that received BoNT/A1 or BoNT/A2 (Figure 6A). Optical density measurements (Figure 6B) showed that in both the ipsilateral and contralateral ventral horns, cSNAP-25 labeling in rats injected with BoNT/A1 was significantly higher than that with BoNT/A2 (*P* < 0.05, Mann–Whitney *U*-test). Thus, it is likely that there exists a wider spread of cSNAP-25 associated with BoNT/A1 than BoNT/A2 in the spinal cord after peripheral muscular application of the toxins.

## Discussion

**Figure 7:**
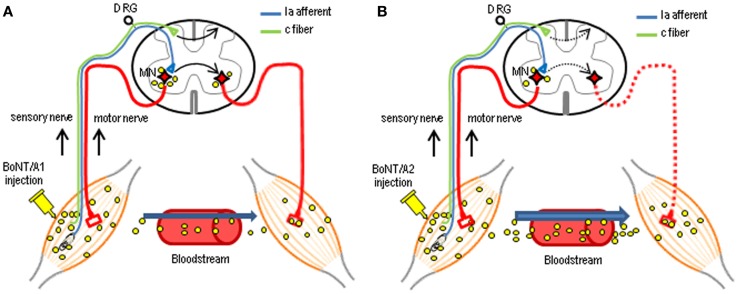
**Possible mechanisms for the central actions of intramuscularly injected BoNT/A in the spinal cord**. Following unilateral intramuscular BoNT/A1 **(A)** or BoNT/A2 **(B)** injection, the catalytically active toxin can be axonally transported to the spinal cord through motor and sensory nerves. Subsequently, the toxin can spread throughout the gray matter of the spinal cord, including the bilateral ventral and dorsal horns, via a transcytosis (cell-to-cell trafficking) mechanism by which a ligand penetrates the neuron at one side, followed by its movement and release at the opposite end, with possible uptake by second-order neurons. Differential delivery routes by which injected BoNT/A1 and BoNT/A2 affect contralateral muscles have also been proposed as BoNT/A1 **(A)** is transported almost equally to the contralateral muscles via this neural pathways and the blood circulation, while BoNT/A2 **(B)** is mainly transported to contralateral muscles via the bloodstream only at higher doses. MN, motoneuron; DRG, dorsal root ganglia.

In this study, we determined the functional consequences of injection of BoNT/A1 or BoNT/A2 into the gastrocnemius muscles of the unilateral hind legs of rats, and the spinal distribution of cSNAP-25 after this injection. CMAP measurements revealed the novel finding that the injected BoNT/A1 or BoNT/A2 exerted paralytic effects on both hind legs. The central actions of intramuscular application of BoNT/A are proposed to occur through axonal and transsynaptic transport (14, 25–28). There is accumulating evidence that like tetanus neurotoxin, BoNT/A can be transported from peripheral neuromuscular junctions to the central nervous system [for review see Ref. (14, 29)]. In a series of experiments (17, 24), we also reported electrophysiological data indicating that BoNT/A1 and BoNT/A2 can be carried from the peripheral to central nervous system and vice versa by dual antero- and retrograde axonal transport through either motor or sensory neurons. Indeed, the present histological results revealed that cSNAP-25 appeared in the bilateral ventral and dorsal horns in distant spinal regions that send efferents to the BoNT/A1- or BoNT/A2-injected muscles. As shown in Figure 7, we suggest that catalytically active BoNT/A1 or BoNT/A2 is axonally transported via peripheral motor and sensory nerves and translocates to the spinal cord, where the toxin spreads through a cell-to-cell trafficking mechanism. In accordance with our previous data that axonal and transsynaptic transport of BoNT/A1 is greater than that of BoNT/A2 (24), the present study also showed a wider spread of cSNAP-25 associated with BoNT/A1 than BoNT/A2 in the contralateral spinal cord, particularly in the ventral horn (see Figure 6). Although it remains unclear how central synapses targeted by BoNT/A after axonal transport and transcytosis are functionally altered, the central actions of transported BoNT/A could improve clinical symptoms by reinforcing the efficacy of peripheral blockade. It is plausible that direct spinal action of BoNT/A results in both motor terminal regeneration and central synaptic reorganization after retrograde transport, so that the supraspinal descending pathways can re-establish contact with lower motor neurons in the spinal cord (15).

The present study showed that unilateral intramuscular BoNT/A1 or BoNT/A2 injection resulted in not only ipsilateral but also contralateral muscular flaccidity. Differential delivery routes by which injected BoNT/A1 and BoNT/A2 affect contralateral muscles have been suggested (17, 24) as BoNT/A1 is transported almost equally to the contralateral muscles via neural pathways and blood circulation, while BoNT/A2 is mainly transported to contralateral muscles via the bloodstream (see Figure 7). This novel evidence might corroborate the present finding that BoNT/A1 injection caused a significant decrease in CMAPs of contralateral muscles associated with an abundance of cSNAP-25 in the contralateral ventral horn, while BoNT/A2 injection did so in despite of a paucity of cSNAP-25 in the contralateral ventral horn. To further elucidate this hypothesis, more precise and quantitative assessments of cSNAP-25 systemic distribution should be performed.

The bilateral muscle relaxation effects seen after unilateral toxin injection may lead to opposite clinical results depending on the somatic symptom distribution of patients. For examples, unilateral toxin injection could be beneficial in patients with bilateral spasticity due to spinal cord injuries but harmful in patients with unilateral spasticity due to forebrain cerebral apoplexy. Experimental evidence from animal models has shown that BoNT/A1 can undergo axonal transport and transcytosis, which results in central effects, particularly when high doses are used (14, 25, 30). Indeed, we here showed that contralateral muscular flaccidity following unilateral, peripheral toxin injection increased in a dose-dependent manner in a dose-dependent manner ranging from 1.7 to 13.6 U. Although the biological effects obtained with the total amount of injected toxin in rats could not easily compared with those in humans, Caleo et al. (14) suggested that the dosage of about 5 U in rats might be almost equivalent to a maximum dose that can be used for the treatment of dystonia and spasticity in patients. Keeping in mind the potential risk due to undesired contralateral central effects of BoNT/A1, the dosage of BoNT/A1 used should be carefully calibrated for each patient. This notion would also apply in the future event of clinical use of BoNT/A2. On one hand, as shown, BoNT/A2 might affect contralateral muscles largely through the bloodstream (17, 24), we also posit that such adverse effects of BoNT/A2 could be easily removed by the injection of A2-antitoxin, suggesting that the usage of BoNT/A2 would be much safer than that of BoNT/A1.

BoNT/A1 can be effectively used to treat some pathological pain conditions in patients (31). Recent reports have shown experimental evidence for central antinociceptive action of peripherally applied BoNT/A1 (32, 33). Our present results also revealed that cSNAP-25 was highly concentrated in the superficial layer of the ipsilateral dorsal horn at the L5 spinal segment after unilateral peripheral injection of BoNT/A1 as well as BoNT/A2. This novel finding could further our understanding of the antinociceptive mechanism(s) of BoNT/A. We speculate that BoNT/A is axonally transported along the peripheral branch of nociceptive sensory neurons (i.e., c-fiber) and then descends into the dorsal horn, where the toxin might exert antinociceptive effects by inhibiting the release of neurotransmitter and neuropeptides (e.g., substance P) from the peripheral branch of primary sensory neurons (34) (see Figure 7). Recent reports have also shown bilateral antinociceptive effects of BoNT/A1 following unilateral, peripheral toxin injection (32, 34, 35). As a possible mechanism by which unilateral BoNT/A1 administration can exert contralateral antinociceptive actions, we suggest that the toxin might spread to contralateral dorsal horn neurons via a crossing fiber mechanism (36) and/or a transsynaptic cell-to-cell trafficking mechanism within the spinal cord. Our assumption is supported by the present immunohistochemical finding that cSNAP-25 was bilaterally distributed in the dorsal horns at the level of L5 (see Figures 4 and 5). This is also confirmed by our previous functional studies, which found that unilateral injection of BoNT/A1 to rat soleus muscle decreased the frequency of glycinergic spontaneous IPSCs in ipsi- and contralateral spinal second-order sensory neurons (24). In addition, BoNT/A2 completely abolished evoked EPSC projecting to spinal sacral dorsal commissural nucleus neurons, one of second-order sensory neurons, in rats (37).

In conclusion, we demonstrated central effects of intramuscularly injected BoNT/A1 or BoNT/A2 in the rat spinal cord. Our results may provide new insight into the clinical effects of peripherally applied BoNT/A in patients with pathological motor and pain conditions.

## Conflict of Interest Statement

The authors declare that the research was conducted in the absence of any commercial or financial relationships that could be construed as a potential conflict of interest. Ryuji Kaji has a patent on A2 botulinum neurotoxin pending (PCT/JP2007/070927).
